# Revisiting customized algorithms for research grade devices

**DOI:** 10.1093/sleep/zsaf011

**Published:** 2025-01-14

**Authors:** Olivia Walch, Michael W L Chee

**Affiliations:** Department of Neurology, University of Michigan, Ann Arbor, MI, USA; Arcascope, Arlington, VA, USA; Centre for Sleep and Cognition, Yong Loo Lin School of Medicine, National University of Singapore, Singapore

The year is 2024. Artificial intelligence is powering drug discovery. ChatGPT is writing essays for 7th graders around the world. And somewhere, a sleep scientist is applying Cole-Kripke [1] or Sadeh [2] algorithms to their activity count data. These classic algorithms have been workhorses since they were first introduced and have had remarkable longevity. But for something more than 30 years old to still be used in the era of modern machine learning and multisensor wearables raises the question: Can we not do better?

There appears to be a limit to the information that can be gleaned from activity counts. A key weakness of actigraphy-only sleep-wake classification is the misidentification of quiet, low-motion wakefulness as sleep. Even for healthy persons, lying in bed using electronic devices can give rise to misclassification of wake as sleep. However, more complex algorithms may not perform significantly better than the classic ones when the inputs are activity counts [3]. Although newer approaches (e.g. leveraging high-resolution acceleration and heart rate) already show superior results, the appeal of backward compatibility is strong: if you use the same methods as a paper from 2002, you can more directly compare to results from 2002. And the most commonly cited reason of all for using classic algorithms or their variants and paying for research grade devices that run them: they are “validated,” implying enduring trustworthiness.

How true is this, though? Hardware has changed in the time since Cole-Kripke [1] or Sadeh [2] were developed: Original accelerometers were single axis systems, while modern ones are all 3-axis with rotation and orientation detection. The accelerometer in the research grade ActiGraph GT9X has poorer ability to infer heart rate from movement than a consumer Apple Watch Series 7, possibly due to a motion artifact in the acceleration signal caused by the GT9X’s design [4]. Firmware updates can also alter the way sensor data are converted into counts, e.g. with newer firmware versions yielding significantly greater counts in the vertical direction [5], which occurs upstream of any algorithm that translates counts into sleep measures, the topic of academic research such as Cepni’s. Such differences by themselves could nullify past validation work.

While updates to firmware and hardware have somewhat stabilized as a result of the sheer number of years actigraphy has been in existence, there remain questions around the implementation of classic algorithms. As Cepni et al. [6] point out, the original algorithms were trained on studies with an AMI Motionlogger that calculated counts through zero-crossings in the acceleration signal. Yet, this definition of a count is different from ones recently disclosed by ActiGraph in their ActiLife software today. ActiGraph alludes to an ambiguous scaling factor for activity counts in the descriptions of the Cole-Kripke and Sadeh algorithms on their website—which one is used in practice? What postprocessing is used? Given these unknowns, then, it is perhaps not surprising that Cepni et al. [6] find that two different implementations of the same algorithm, the ActiLife version and their open source GGIR version, give statistically distinct sleep metrics when fed identical acceleration data. In the same vein, a recent analysis of actigraphic data from two popular research grade devices collected from a longitudinal ageing study cautioned that, apart from total sleep time, sleep variables were not comparable across these devices [7]. Research grade is not the panacea that some might believe it to be.

Desire to improve sleep and wake classification using wearable devices has led to two research verticals: (1) evaluating the performance of consumer wearables with proprietary algorithms, such as Fitbit, Oura, and the Apple Watch, and (2) developing open source models based on raw or minimally processed data, as Cepni et al. do. Validation studies of closed-source commercial wearables have value and have recently shown that multisensor consumer devices outperform research actigraphs in two-stage sleep classification [8, 9]. However, there is valid concern that the manufacturer can change their algorithm with little or no warning, possibly halfway through a study materially affecting results. In 2021, for example Apple changed their heart rate variability algorithm so that the same data, from the same collection period, pulled from the device at two time points could be markedly different [10].

Yet, critics of consumer devices should be aware that firmware and data interpretation algorithms can also change in research devices, leading to similar problems as those encountered with consumer ones. Notably, few, if any, older papers record firmware or software versions used. Furthermore, as updates generally correct errors, it may not be advantageous to stick to an older system for backward compatibility. In light of this, perhaps what we really mean by “validated” when we use it to praise research grade devices is that they do not change very much. This is a weak point in favor of their use.

It only makes sense to completely close the door to consumer devices if there is no way to address the black-box algorithm problem. This may be changing. Oura has added a feature to “freeze” firmware when devices are used in research studies, and it indicates months ahead of time when a major software update is to be implemented. Longitudinal data with research grade actigraphs have typically been collected over shorter ~3 months time frames and often only a week for an individual. Months-ahead notice should provide ample time for researchers to plan for adjustments.

For studies where data collection spans years across different participants, genomics, and brain imaging fields deploy statistical and deep learning methods for data harmonization, such as ComBat, that our field can learn from. Brain imaging researchers also evaluate representative participants on old and new algorithms to allow some mapping of results across changes in scanners or firmware versions, in a form of benchmarking [11]. Similar approaches could be used in the sleep field with older and newer versions of consumer sleep algorithms.

The second research vertical, open source algorithms of the kind developed by Cepni et al. [6] and other research groups [12], offers a solution to the black-box algorithm problem while not tying us to historical algorithms. The performance of new iterations of such algorithms can be benchmarked against open datasets. Their versions can be tagged with release notes and clear identifiers, streamlining backward compatibility. The authors of Cepni et al., having made the GGIR package which is currently in use by many groups, should be praised for their work building the code and achieving that adoption. The next sleep classification algorithm to achieve influence at the level of Cole-Kripke over the last 30 years could be open source, especially if it leverages recent developments in sensor technology and machine learning techniques.

Groups developing these algorithms must acknowledge, however, that the questions around differential sensor performance and data artifacts that exist for companies are just as relevant for open source approaches that aim to be device-agnostic. There are also challenges with implementation: Provision of raw accelerometer data reduces battery life, involves long wireless data transfer times, and adds costs which may be acceptable for researchers but not for consumers. Obtaining large high-quality training data across diverse ages, use contexts and medical conditions takes money and dedicated resources. It is not enough to post code online once and declare victory—for an open source algorithm to have a lasting impact, it needs to be broadly and easily usable, which requires constant upkeep. GitHub contains many well-intentioned open source projects that no longer function because the libraries they were built on have changed so dramatically. Additionally, few scientific code graphical user interfaces can hold a candle to those of commercial products.

The sleep field is full of lots of talented people. We should give kudos when companies, such as ActiGraph, make the move to open source their current activity counts algorithm, as they have done on their GitHub page. We should give kudos to the authors of Cepni et al. for their commitment to developing in the open and pushing for adoption of their algorithms. However, there are truths we must acknowledge. Using a “research grade” device does not magically make data more accurate or immune to concerns about black-box algorithm firmware and software updates. Basing algorithms on activity counts may make their performance backward compatible, but it does so at the cost of limiting their performance. Open source algorithms that use new streams of input data—such as heart rate or high-resolution acceleration data—can move us beyond the classic algorithms, but their codebases take continuous effort and resources to support.

We are highly confident that in the years ahead, new approaches will rise to take us from Cole-Kripke [1] or Sadeh [2] and into the future. Improvements to sleep-wake measurements are possible, with a variety of devices, but only if we recognize the many challenges inherent to transforming sensor data into clinically relevant outputs, embrace fresh approaches, and partner with industry to find mutually beneficial solutions.
